# Highlighting the effects of high-intensity interval training on the changes associated with hypertrophy, apoptosis, and histological proteins of the heart of old rats with type 2 diabetes

**DOI:** 10.1038/s41598-024-57119-6

**Published:** 2024-03-26

**Authors:** Mohammad Rami, Amirhossein Ahmadi Hekmatikar, Samaneh Rahdar, Sayed Shafa Marashi, D. Maryama Awang Daud

**Affiliations:** 1 Department of Sport Physiology, Faculty of Sport Sciences, Shahid Chamran University of Ahvaz, Ahvaz, Iran; 2 Department of Physical Education and Sport Sciences, Faculty of Humanities, Tarbiat Modares University, Tehran, 10600 Iran; 3 Department of Basic Sciences, Histology Section, Faculty of Veterinary Medicine, Shahid Chamran University of Ahvaz, Ahvaz, Iran; 4 Department of Sport Physiology, Faculty of Sport Sciences, Shahid Chamran University of Ahvaz, Ahvaz, Iran; 5 Health Through Exercise and Active Living (HEAL) Research Unit, Faculty of Medicine and Health Sciences, Universiti Malaysia Sabah, Kota Kinabalu, 88400 Sabah, Malaysia; 6 Department of Biomedical Sciences, Faculty of Medicine and Health Sciences, University Malaysia Sabah, Jalan UMS, Kota Kinabalu, 88450 Sabah, Malaysia

**Keywords:** Type 2 diabetes mellitus, High-intensity interval training, Aging, Cardiovascular diseases, Endocrine system and metabolic diseases, Ageing

## Abstract

T2DM is known to cause disturbances in glucose homeostasis and negative changes in the heart muscle, while aging and diabetes are recognized risk factors for CVD. Given this, our study aims to investigate a method for controlling and managing CVDs induced by T2DM in elderly populations. To achieve this, we categorized 40 rats into 5 groups, including HAD (n = 8), HA (n = 8), AD (n = 8), AHT (n = 8), and ADT (n = 8). The exercise protocol consisted of eight weeks of HIIT (three sessions per week) performed at 90–95% of maximal speed. Following cardiac tissue extraction, we assessed the levels of IGF-1, PI3K, and AKT proteins using Western blot technique, and analyzed the histopathological variations of the heart tissue using H&E, Sudan Black, and Masson’s trichrome tissue staining. The histological findings from our study demonstrated that T2DM had a significant impact on the development of pathological hypertrophy and fibrosis in the heart tissue of elderly individuals. However, HIIT not only effectively controlled pathological hypertrophy and fibrosis, but also induced physiological hypertrophy in the AHT and ADT groups compared to the HA and AD groups. Results from Sudan Black staining indicated that there was an increase in lipid droplet accumulation in the cytoplasm of cardiomyocytes and their nuclei in the HA and AD groups, while the accumulation of lipid droplets decreased significantly in the AHT and ADT groups. In both the AHT group and the ADT group, a single HIIT session led to a reduction in collagen fiber accumulation and fibrotic frameworks. Our research also revealed that diabetes caused a significant elevation in the levels of IGF-1, PI3K, and AKT proteins, but after eight weeks of HIIT, the levels of these proteins decreased significantly in the training groups. Overall, our findings suggest that HIIT may be a suitable non-pharmacological approach for improving histological and physiological changes in elderly individuals with T2DM. However, we recommend further research to examine the impact of HIIT training on both healthy and diseased elderly populations.

## Introduction

Today, T2DM is recognized as one of the most common metabolic disorders in the world, alongside other dangerous T2DM. This condition arises due to faulty insulin secretion by pancreatic beta cells and impaired responsiveness of insulin-sensitive tissues to insulin^1^. T2DM is a global chronic disease burden in aging societies^2^. Although type 2 diabetes can be diagnosed in early stages, its complications, particularly cardiovascular issues, tend to be more prevalent during middle age^2^. Therefore, it comes as no surprise that diabetes is increasingly becoming a leading cause of mortality^1,3^. The prevalence of T2DM is increasing day by day due to the mechanization of living standards, decreased PE, and unhealthy diets. In 2019, 463 million adults worldwide, which accounts for 9.3% of the population, suffered from T2DM, and this number is expected to rise to 578 million (10.2%) by 2030 and 700 million (10.9%) by 2045^4^. Understanding some of the cellular mechanisms underlying this disorder can be helpful in developing appropriate treatment strategies.

Current research has focused on understanding how T2DM contributes to CVD and mortality, with cardiac fibrosis emerging as a key factor^5^. Rami et al.^6^ reported that rats with T2DM had significantly more cardiac fibrosis than the healthy control group^6^. MF is strongly associated with CVD events, including HF, CAD, AF, and PAD^7^. MF occurs when there is an excessive and disproportionate increase in collagen concentration in the extracellular matrix of the myocardium^5,7^, which impairs impulse propagation and can lead to arrhythmic events and conduction abnormalities^5,7^. In addition to cardiac fibrosis, another notable change observed in T2DM is cardiac apoptosis, which is the result of cardiomyocyte apoptosis leading to cell loss, reducing the contractile function of the heart and ultimately promoting cardiac regeneration^8^. Studies have shown that apoptosis damages the heart during T2DM through multiple upstream signaling pathways^6,8^. Along with apoptotic^6,8^ and fibrotic^6,8^ changes due to T2DM, hypertrophic changes in the heart have also recently attracted researchers’ attention^9^. Hypertrophic changes in the heart of patients with T2DM can lead to pathological enlargement, CAD, systole and diastole dysfunction, and cardiac arrhythmia^6,9,10^. These pathological and histological changes that occur in the heart of patients with T2DM^5–10^ can originate from primary physiological changes involving various mechanisms, with one of the most important being the AKT/PI3K/IFG-1 pathway^11,12^. IGF-1 is a polypeptide growth factor with a structure comparable to insulin that plays a critical role in maintaining cardiac physiology homeostasis^11^. However, the exact relationship between IGF-1 and CVD remains highly controversial due to limited and conflicting findings. Individuals with higher-than-median insulin levels and high levels of free IGF-1 have a higher chance of developing T2DM, while those below the median have a lower risk^11^. However, one study found no association between IGF-1 levels and T2DM^13^. The negative changes of IGF-1 in T2DM can lead to CVD problems through two perspectives, namely atherosclerosis, inflammation, vasodilation, cardiac apoptosis, and autophagy, and by affecting the PI3K/Akt cascade^11,12^. Activation of the IGF-1 receptor initiates a cascade of reactions with tyrosine kinase-mediated phosphorylation of the IRS-1, an adapter protein that provides a binding site for PI-3 kinase, which activates Akt^11,12^. PI3K/AKT plays an essential role in the pathological processes leading to atherosclerosis, starting from the formation of atherosclerotic plaques^14^. Thus, dysfunction of IGF-1 in T2DM patients can lead to the disruption of PI3K/AKT signaling cascades, which can ultimately affect both the histological and pathological changes^5–10^ of the heart and can lead to physiological disorders of the heart^11–14^.

The importance of medication in the treatment and management of T2DM cannot be overlooked. However, due to the potential side effects and high costs of drug therapy^15^, PE has gained significant recognition as a free and effective treatment option for this disease^16^. Exercise physiologists have been studying optimal PE strategies for the treatment and management of T2DM in patients with CVD for many years. They emphasize the importance of PE due to its effects on cellular and molecular mechanisms^17^. The researchers concluded in their findings that sustainable exercise interventions among elderly individuals can effectively lower blood glucose levels, enhance cardiovascular function, and ultimately contribute to increased life expectancy among elderly individuals with diabetes^18^. Among the various PE strategies available, HIIT has recently gained special attention due to its high intensity and significant histological, pathological, and physiological effects^6^. In a study similar to the present study, Rami et al.^6^ reported that HIIT can be an effective strategy for reducing cardiomyopathy in rats with T2DM^6^. The researchers found that HIIT led to significant improvements in weight, blood glucose levels, histopathology, hypertrophy, Purkinje fibers, collagen area around the heart tissue’s arterial vessels, fibrosis, apoptosis, and antioxidant changes and oxidative stress in T2DM rats^6^. These findings were confirmed by a study conducted by Chavanelle et al. (2017), which demonstrated that HIIT had a more positive effect on blood glucose and heart structure changes in T2DM rats compared to moderate-intensity training^19^. Additionally, previous studies have indicated that HIIT yields greater effects compared to MDT on PI3K/AKT and IGF-1 pathways, leading to positive physiological changes in T2DM patients^20,21^. As this effect manifests within a short duration, some studies have suggested the application of h HIIT for other age groups, including elderly individuals with T2DM^22,23^.

Since the prevalence of acute and chronic diseases such as CVD, T2DM and infections increases with the aging process. In addition, the increasing prevalence of T2DM and its link to CVD underscores the need to comprehend the cellular mechanisms at play in this condition. Concurrently, examining the histological, pathological, and physiological changes in T2DM patients with CVD can help us fathom the altered mechanisms of this disease and pave the way for potential treatment solutions. Researchers widely acknowledge that the most prominent changes in this regard are cardiac fibrosis, apoptosis, and hypertrophy, along with the IGF-1/PI3K/AKT pathway. Given the fact that HIIT yields positive results within a short duration, this study in line aligns with previous research conducted on young individuals with T2DM^6,19^. While earlier studies have highlighted the suitability of HIIT for young adults, the main hypothesis of this study was to specifically address the suitability of HIIT for older adults with T2DM. The findings of this study will serve as a guiding light for future research, enabling researchers to develop sustainable exercise protocols for this population. Thus, the current study aimed to investigate the effect of HIIT on histological changes and protein content related to hypertrophy and apoptosis of heart tissue in aged rats with T2DM.

## Material and methods

### Animals

**Figure1:**
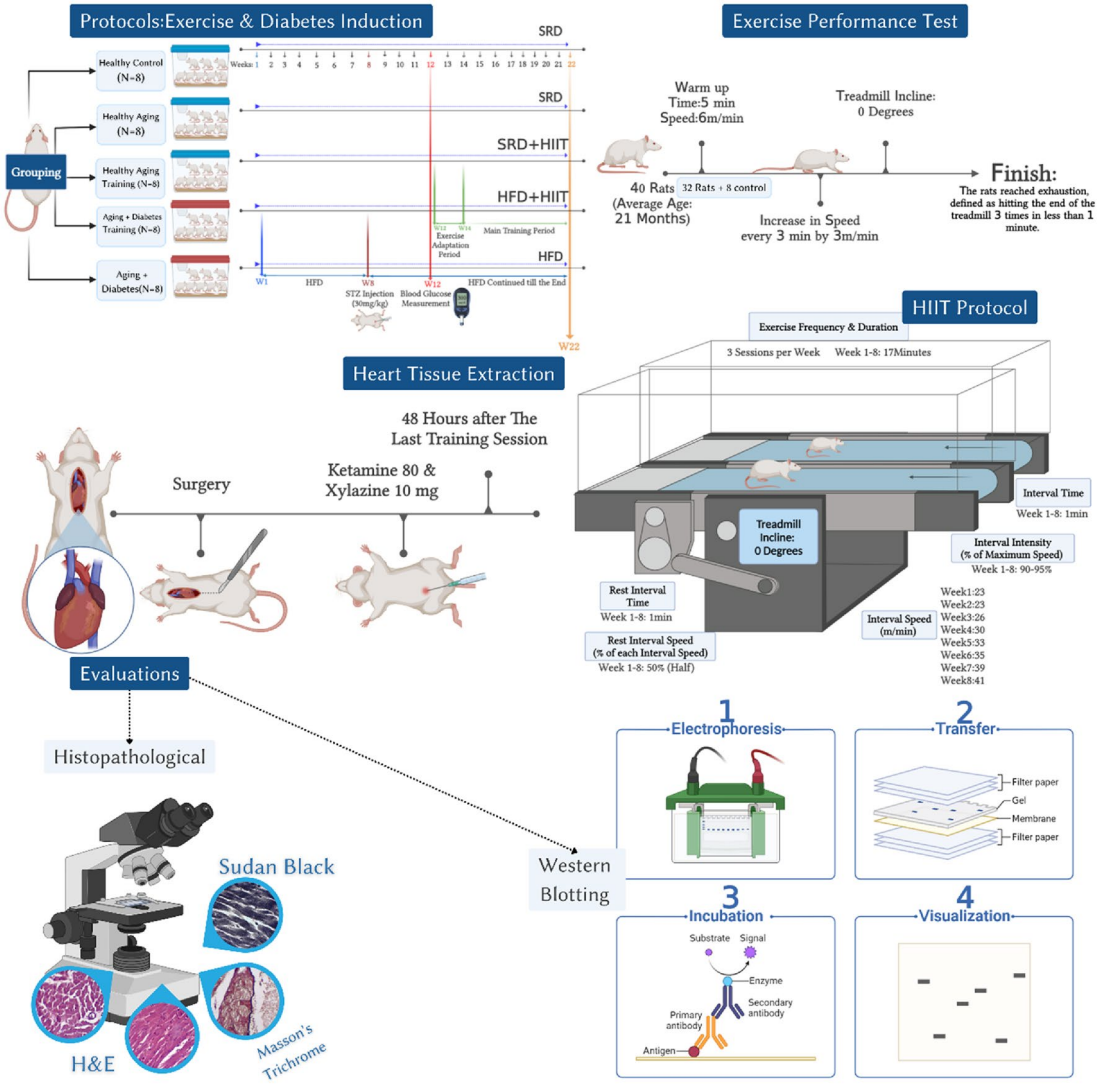
Schematic design of different stages of diabetes induction, HIIT protocol, heart tissue extraction and histological and molecular evaluations. *SRD* Standard rodent diet; *HIIT* High-Intensity Interval Training; *HFD* High Fat diet; *STZ* Streptozotocin; *H&E *Hematoxylin–eosin staining.

All procedures of the present study were conducted in accordance with the Guide for the Care and Use of Laboratory Animals, Eighth Edition (2011). Ethical principles in working with animals were strictly followed, and the study was approved by the Animal Research Ethics Committee of Shahid Chamran University of Ahvaz (EE/1401.2.24.222565/scu.ac.ir). All experiments were performed in accordance with ARRIVE guidelines. Thirty-two male Wistar rats weighing between 400 and 425 g and with an approximate age of 20 to 22 months and eight rats in the control group were 10 weeks old and weighed about 180 to 200 g were obtained from the Animal Care Center of Shahid Chamran University of Ahvaz and used in this research. They were housed in special plexiglass cages under controlled environmental conditions with an average temperature of 22 ± 1.4 °C, 50 ± 4% humidity, and a 12 h:12 h light–dark cycle. The rats had free access to food and water dedicated for laboratory animals. After acclimation to the laboratory environment, the male rats were randomly assigned to one of five groups: HAD (n = 8), HA (n = 8), AD (n = 8), AHT (n = 8), and ADT (n = 8) (see Fig. 1). After inducing diabetes, rats in the AHT and ADT groups followed an 8-week exercise protocol in the exercise room of the animal house of the Faculty of Veterinary Medicine of Shahid Chamran University of Ahvaz.

### Diabetes induction

To induce diabetes, we followed the protocol provided by Zhang et al.^24^ and Liu et al.^25^. We administered a high fat diet (HFD) for 8 weeks, which consisted of 60% energy from fat, 20% from carbohydrates, and 20% from protein (21.5 kcal/g). In the fourth week, we intraperitoneally injected the HFD/STZ-induced diabetic groups with a low dose of STZ (30 mg/kg) (Sigma, Germany). After four weeks, we measured the blood glucose levels using a glucometer (Roche Diagnostics K.K., Japan). We included subjects with blood sugar levels greater than 300 mg/dl in the study as diabetic samples^26^. Rats in the non-diabetic groups were injected with an equivalent volume of sodium citrate buffer (0.25 ml/kg). After confirming diabetes induction, we fed aged rats with HFD/STZ-induced T2DM a high-fat diet consisting of 55% fat, 31% carbohydrate, and 14% protein. Animals in the HA groups were fed a standard rodent diet, which consisted of 10% fat, 75% carbohydrate, and 15% protein (supplementary material and Fig. 1).

### Exercise performance test and HIIT protocol

To acclimatize the rats to the conditions of HIIT, they underwent a 14 day training regimen on a treadmill set to a speed range of 10–30 m/min and 0 degree. Prior to initiating the primary training protocol, the rats underwent an incremental test to determine their maximum speed, which was performed after the familiarization period^27^. The maximum speed was determined by gradually increasing the treadmill speed by 3 m/min every 3 min, following a 5 min warm-up run at a speed of 6 m/min and 0 incline. The test concluded when the rat reached the point of exhaustion, defined as hitting the end of the treadmill three times in less than one minute^27^. To adhere to the principle of progressive overload, a maximum running speed test was performed at the onset of each week. After determining the maximum speed, the primary training protocol was initiated. The training groups underwent an 8 week program, with three weekly sessions, each comprising nine intervals of 1 min at an intensity of 90–95% of the maximum running speed. Following each interval, the rats had 1 min of active rest at 50% of the maximum running speed^27^. At the start and end of each training session, the rats underwent a 4 min warm-up and cool-down period, respectively, at an intensity of 45–55% of the maximum speed. The control groups did not participate in any exercise program during the HIIT protocol (see Fig. 1).

### Heart tissue extraction

At the conclusion of the eighth week and 48 h following the last training session, the rats were anesthetized through intraperitoneal injection using a combination of 80 mg ketamine and 10 mg xylazine. The heart tissue was then extracted under sterile conditions and promptly transferred to a − 70 °C freezer (model 88FD-2-93-A, Iran Madas Company).

### Antibodies

Anti-IGF-1 antibody (ABIN5066654), Anti- Akt1 (A-11): sc-377457 (SANTA CRUZ), Anti-PI 3 Kinase catalytic subunit gamma/PI3K-gamma antibody ab154598 (abcam), Mouse anti-rabbit IgG-HRP: sc-2357 (SANTA CRUZ) and HPRT (HPRT Antibody (F-1): sc-376938, SANTA CRUZ).

### Western blot

In order to measure proteins, a lysis buffer was prepared. Tissue samples were subsequently frozen at − 70 °C to prepare tissue homogenate and conduct a western blot test. The amount of protein in the tissue homogenate was determined using the Bradford method, whose analysis method is provided in Supplementary Material 2. Polyacrylamide gel electrophoresis with SDS was then used, with the procedure, buffers, and solutions required for SDS-PAGE and different steps of polyacrylamide gel electrophoresis with SDS being detailed in. Following electrophoresis, blotting, blocking, incubation, and emergence were carried out, with the buffers and solutions, transfer steps (blotting), method, and blocking action by blocking buffer (blocking) all being detailed in Supplementary Material 2. In order to observe the ladder bands and validate the accuracy of the western blot protocol setup, the sections corresponding to the ladder were cropped onto nitrocellulose paper prior to incubating the primary and secondary antibodies. After several runs, high-quality figure were selected and low-quality figure were removed.

To examine changes, Hematoxylin–eosin (H&E)^6,28^ staining was utilized to examine histological changes in cellular and structural details. Black Sudan histochemical staining was used to observe lipid accumulation in the cytoplasm of cardiomyocytes^6,28^. Masson’s Trichrome staining^6,29^ was used to detect collagen fibers in tissues, with the staining and preparation method being detailed in Supplementary Material 3.

### Data analysis and statistics

The normality of the data was assessed using the Shapiro–Wilk test, while the homogeneity of variances was evaluated using Levene’s test. Changes in glucose and weight at various stages of exercise were investigated using a mixed ANOVA test (composite variance analysis). To compare the means of the studied variables, a one-way ANOVA test was performed, followed by Tukey’s test as a post-hoc analysis. The significance level was set at P ≤ 0.05. All statistical analyses were conducted using SPSS software version 25.

### Ethics statement

The animal study was reviewed and approved by Shahid Chamran University of Ahvaz: EE/1401.2.24.222565/scu.ac.ir.

## Results

In this present study, we have conducted an investigation into the histopathological and histomorphometric alterations in the cardiac tissue of diabetic rats subsequent to HIIT. To assess the impact of HIIT on the heart, we subjected the myocardial tissue of non-diabetic and diabetic mature rats to histological examination using a light microscope. Hematoxylin and eosin (H&E), Masson’s trichrome, and Sudan black stains were utilized to stain the myocardial tissue.

### Changes in average weight and blood glucose

**Figure 2:**
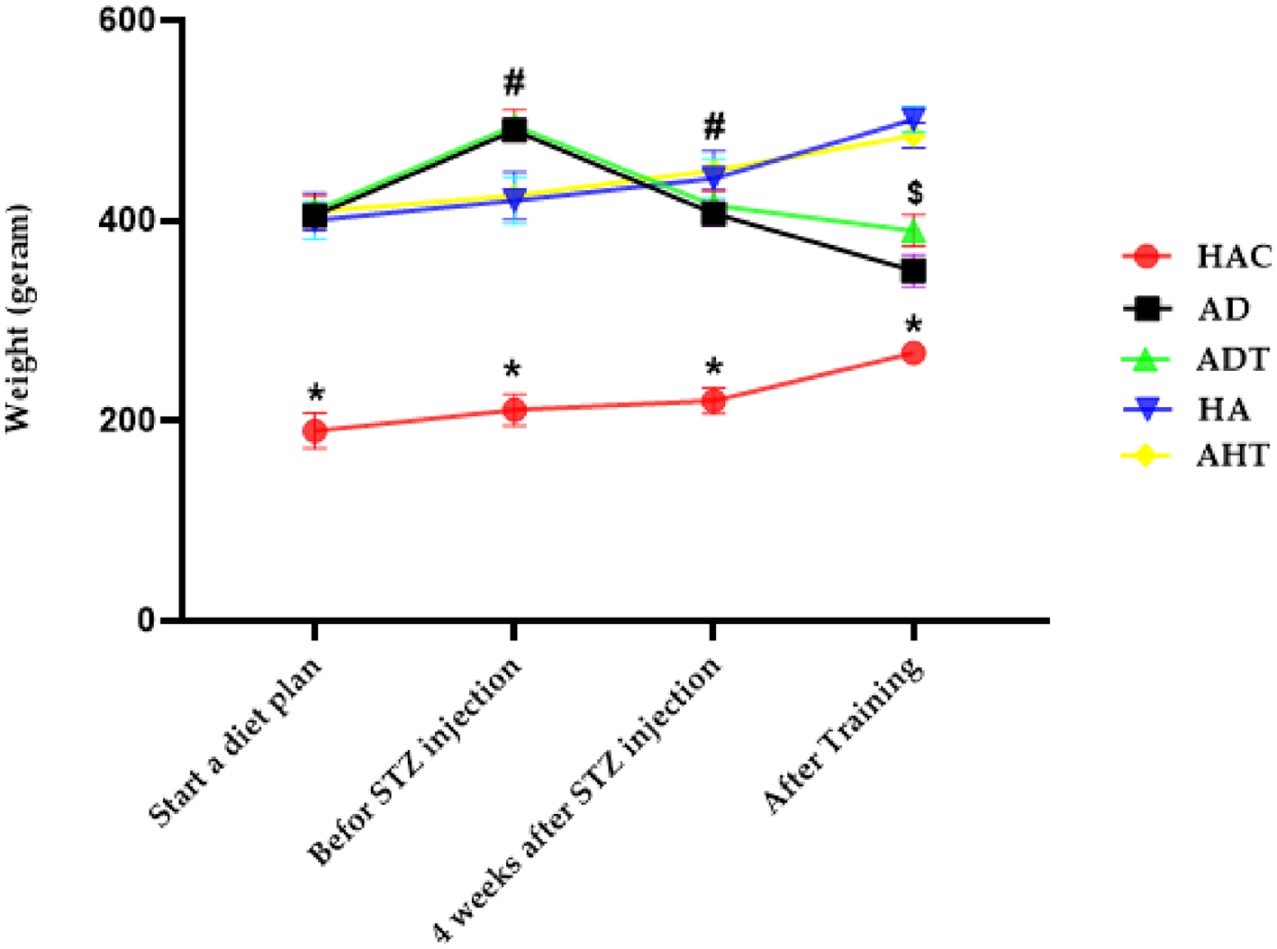
Changes in weight in different groups and in different stages of the exercise protocol. Asterisk Significant difference in the weight of HAC group in compare to other groups in the all stage of protocol. Hash significant difference in weight of AD and ADT groups with other groups in before STZ injection and 4 weeks after STZ injection phases. Dollar Significant difference in weight of ADT group with AD and other groups in After Training phase. The significance level is P < 0.05.

Figure 2 illustrates the alterations in mean body weight and blood glucose levels among various groups. The findings of the mixed ANOVA test revealed that the body weight of rats in the diabetic groups exhibited a significant increase from the initiation of the high-fat diet until the stage preceding STZ injection. Subsequently, the body weight of the rats decreased significantly four weeks after STZ injection (P < 0.001). Additionally, the ADT group demonstrated a lesser reduction in body weight compared to the AD group upon completion of the exercise protocol (P < 0.001) (Fig. 2).

**Figure 3:**
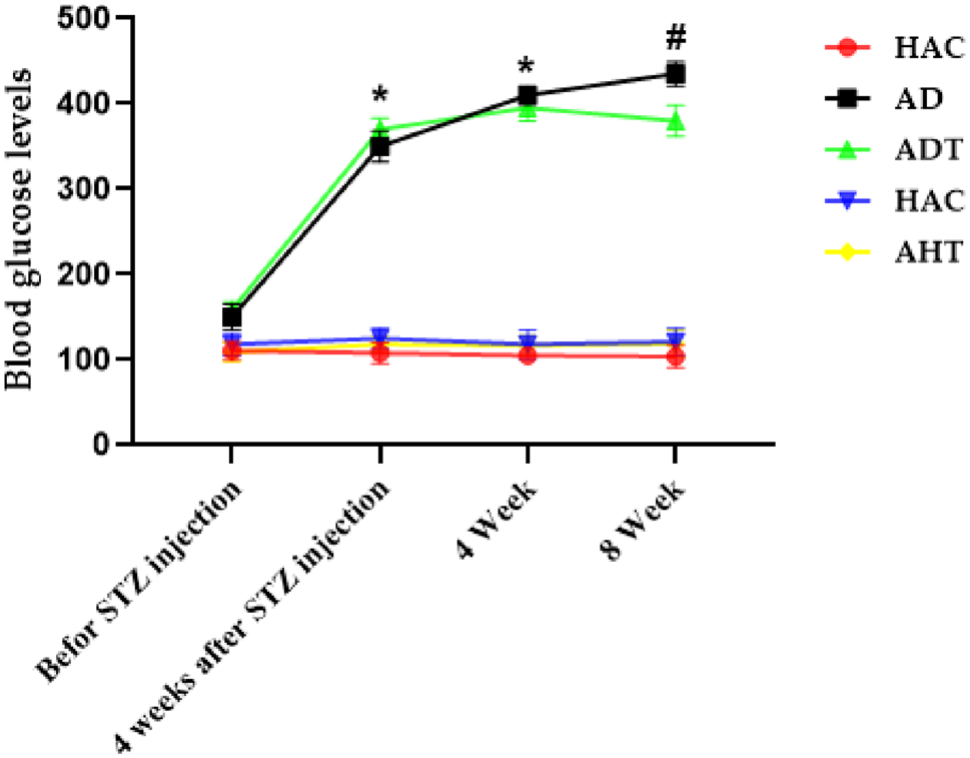
Changes in blood glucose levels in different groups and in different stages of the exercise protocol. Asterisk Significant difference in blood glucose levels of AD and ADT groups with other groups in 4 weeks after STZ injection and week 4 phases and Significant difference in blood glucose levels of AD and ADT groups compared to before STZ injection phase. Hash significant difference in blood glucose levels of AD with ADT d and other groups in week 8 phase. The significance level is P < 0.05.

Moreover, the outcomes of the study indicated that the blood glucose levels increased significantly four weeks after STZ injection and fourth week of exercise in the AD and ADT groups, but decreased significantly in the ADT group in contrast to the AD group upon completion of the exercise protocol in the eighth week compared to fourth week, (P < 0.001) (Fig. 3).

### Histological changes and hypertrophy of heart tissue

**Figure 4:**
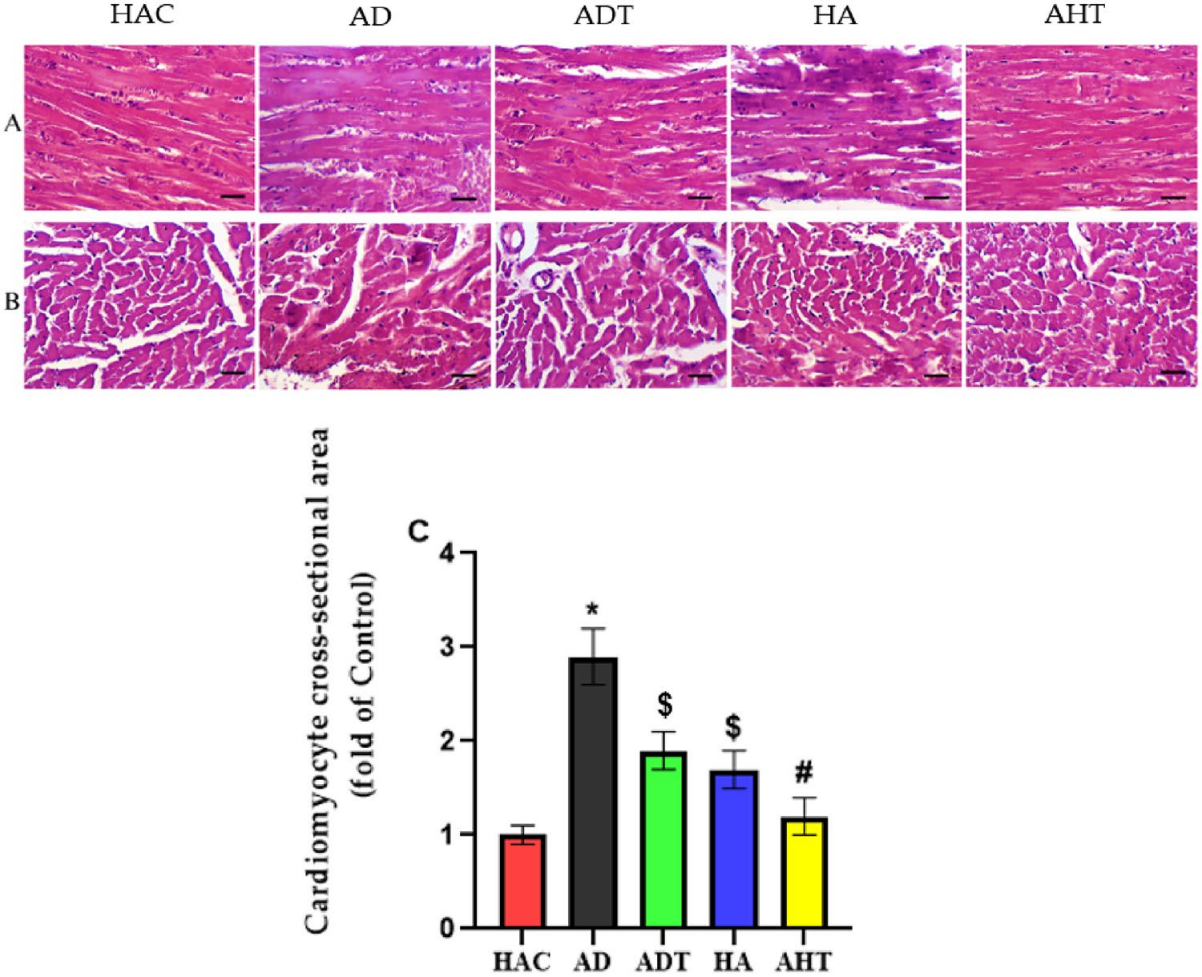
Results of histology and histomorphometry of heart tissue with H&E staining. (**A**) longitudinal section and (**B**) transverse section. All images represent × 40 magnification and scale bars represent 20 μm. (**C**) Diagram of the average cross-sectional area of cardiomyocytes of heart tissue. Asterisk Significant difference with all groups (P < 0.001). Dollar Significant difference with HAC, AD and AHT group (P < 0.05). Hash Significant difference with HA, ADT and AD groups (P < 0.05).

The findings obtained from H&E staining indicated that the heart tissue of the HAC exhibited a normal myofibril structure with nuclei located in the center in both longitudinal and transverse sections, in contrast to the HA group and the AD group (Fig. 4A,B). The AD group, on the other hand, revealed damaged and anomalous myofibrils, indistinct nuclei, augmented collagen connective tissue, and bleeding in both longitudinal and transverse sections (Fig. 4A,B). The results of the study also indicated the disturbance of cardiomyocytes and a substantial increase in the interstitial space in the HA groups. H&E staining illustrated an enlargement in the cross-sectional area of cardiomyocytes in the hearts of aged rats, indicating the occurrence of pathological hypertrophy (Fig. 4A,B). Conversely, in the heart tissue of AHT rats and ADT rats, the damage to the myofibrils of the heart tissue decreased, the cell nuclei became more distinct, the bleeding in the interstitial tissue decreased, and the amount of connective tissue (Collagen) decreased. In fact, the cardiomyocytes in the HIIT groups exhibited a regular arrangement, a normal interstitial space, and the heart tissue of the rats in the AHT and ADT groups displayed physiological hypertrophy compared to the HA and AD groups (Fig. 4A,B). The histomorphometric findings of the heart tissue indicated that the mean cross-sectional area of cardiomyocytes in the AD group was significantly greater than that of the HAD and HA groups (P < 0.001) (Fig. 4C). Furthermore, the outcomes of the study revealed that after a period of HIIT exercise in the AHT and ADT groups, the pathological hypertrophy was effectively managed (P < 0.05). The area of cardiomyocytes in each image was measured using Image J software, and the averages were presented as a fold of control for further analysis. Histomorphometri results of the average cross-sectional area of myocardial cells has been shown in (Fig. 4C).

## Examination of cardiac tissue fibrosis

**Figure 5:**
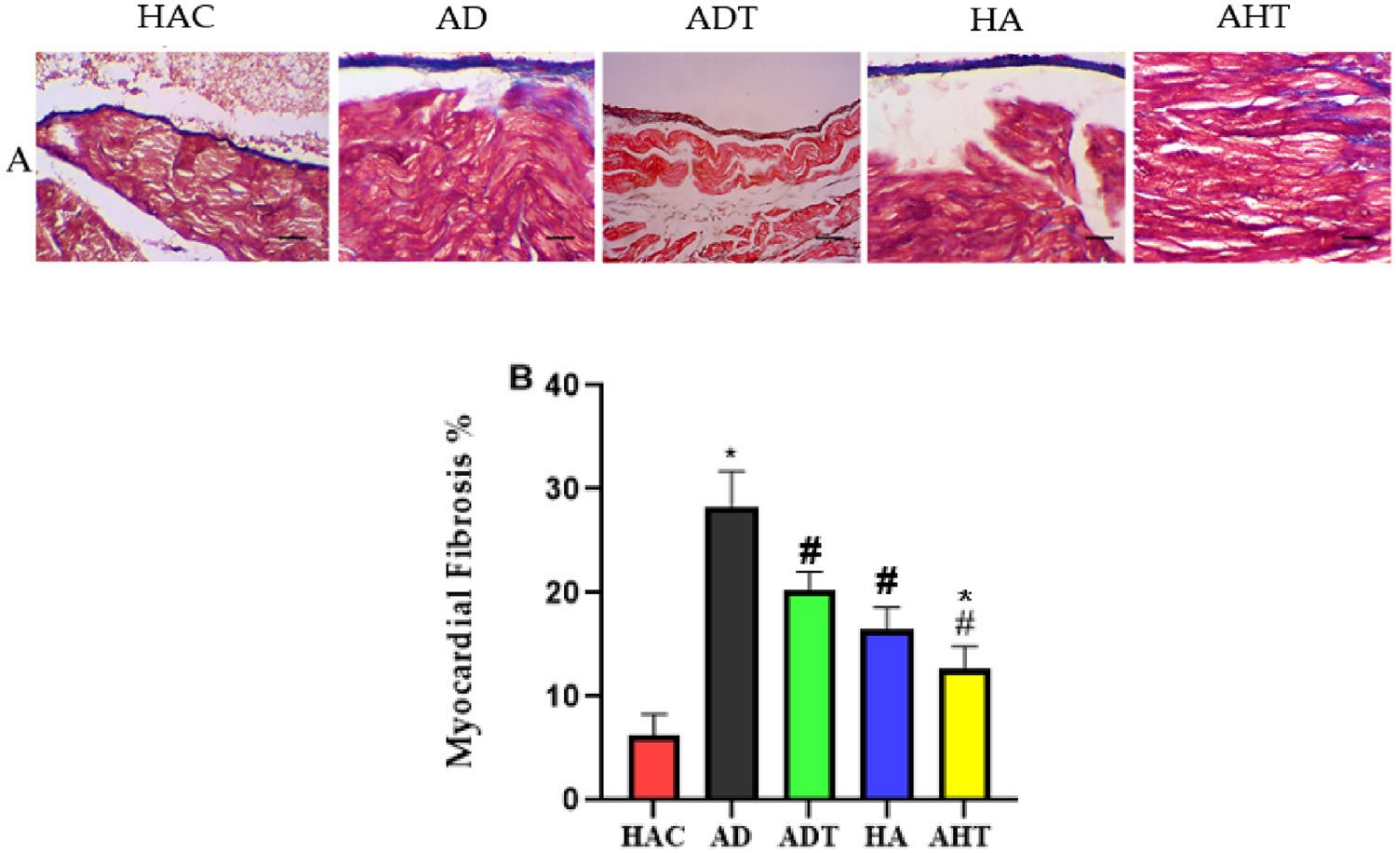
(**A**) Masson’s trichrome staining results of heart tissues of rats of different groups following HIIT in order to investigate cardiac tissue fibrosis. All images represent × 10 magnification and scale bars represent 50 µm. (**B**) The graph of fibrosis changes in the heart tissue of elderly rats, which was done as a percentage of the positive area of fibrosis in the entire cross-sectional area, using Image J software. Asterisk Significant difference with all groups (P < 0.001). Hash Significant difference with the HAC, AD and AHT groups (P < 0.001).

To evaluate changes in cardiac tissue caused by aging, we examined the cross-sectional area of the heart and interstitial fibrosis. We assessed the area of cardiac tissue fibrosis by analyzing extracellular matrix and collagen accumulation using Masson’s trichrome staining, which revealed itself in the form of collagen scaffolds. Masson’s trichrome staining analysis revealed a significant increase in interstitial fibrosis in the hearts of aged rats (P < 0.001). In addition, we found that AD rats exhibited more collagen deposition in the myocardium compared to HAC and HA rats (P < 0.001). Furthermore, the results indicated that the accumulation of collagen and interstitial fibrosis in the heart tissue of rats in the AHT group was lower than that of the AD group (Fig. 5A,B).

### Investigating the accumulation of lipid droplets in the heart tissue

**Figure 6:**
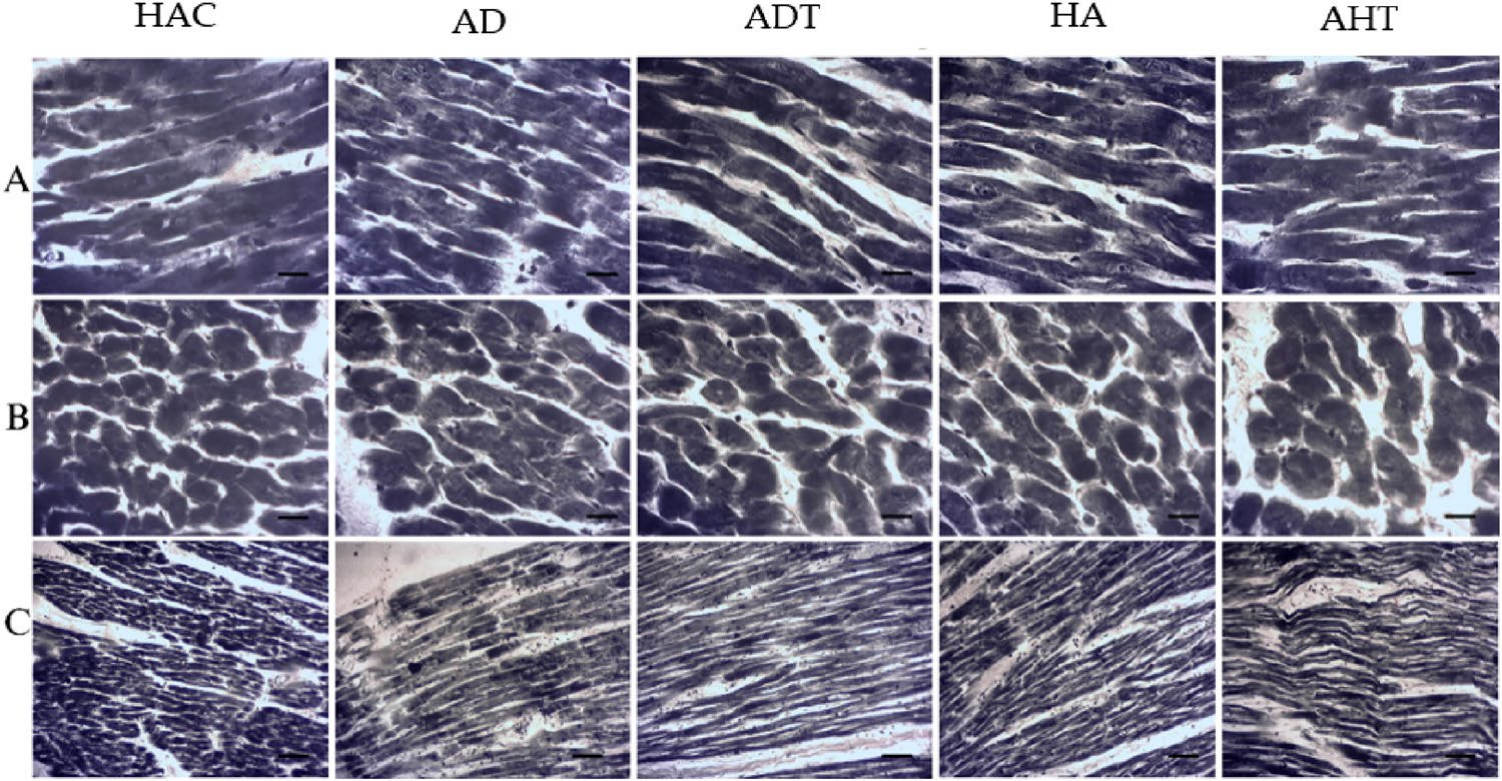
The amount of accumulation of lipid droplets in the heart tissue in different groups. (**A**) Longitudinal section and (**B**) Transverse section. All images represent × 40 magnification and scale bars represent 20 μm. In the healthy aging training group and the diabetes training group, the reduction of the reaction to Sudan Black staining is significant due to the decrease in the accumulation of lipid droplets in the cytoplasm. In Fig. (**C**), changes in the accumulation of collagen fibers and fibrous scaffolds can be seen in different groups. All images represent × 10 magnification and scale bars represent 50 µm.

Sudan Black histochemical staining was used to investigate lipid accumulations in the cytoplasm of cardiomyocytes. Lipid accumulations were identified by the presence of black seeds in the cytoplasm of cardiomyocytes. Cardiomyocytes and their nuclei in the HAC group and the AHT group displayed normal structure and did not show any reaction to Sudan Black staining in longitudinal and transverse sections (Fig. 6A,B). However, in the HAC and ADT groups, there was an increase in the accumulation of lipid droplets in the cytoplasm of cardiomyocytes and their nuclei in longitudinal and transverse sections (Fig. 5A,B). In contrast, the accumulation of lipid droplets decreased significantly in the AHT and ADT groups (Fig. 6A,B) after a period of HIIT exercise. Moreover, Sudan Black staining in the longitudinal section of cardiac tissue revealed a significant increase in collagen fibers, fibrous scaffolds, and the infiltration of mononuclear cells (infiltration of inflammatory cells) in the HAC and AD groups. However, in both the AHT and ADT groups, a period of HIIT exercise resulted in less accumulation of collagen fibers, fibrous scaffolds, and mononuclear cells (Fig. 6C).

### Evaluating the changes in the content of proteins

**Figure 7:**
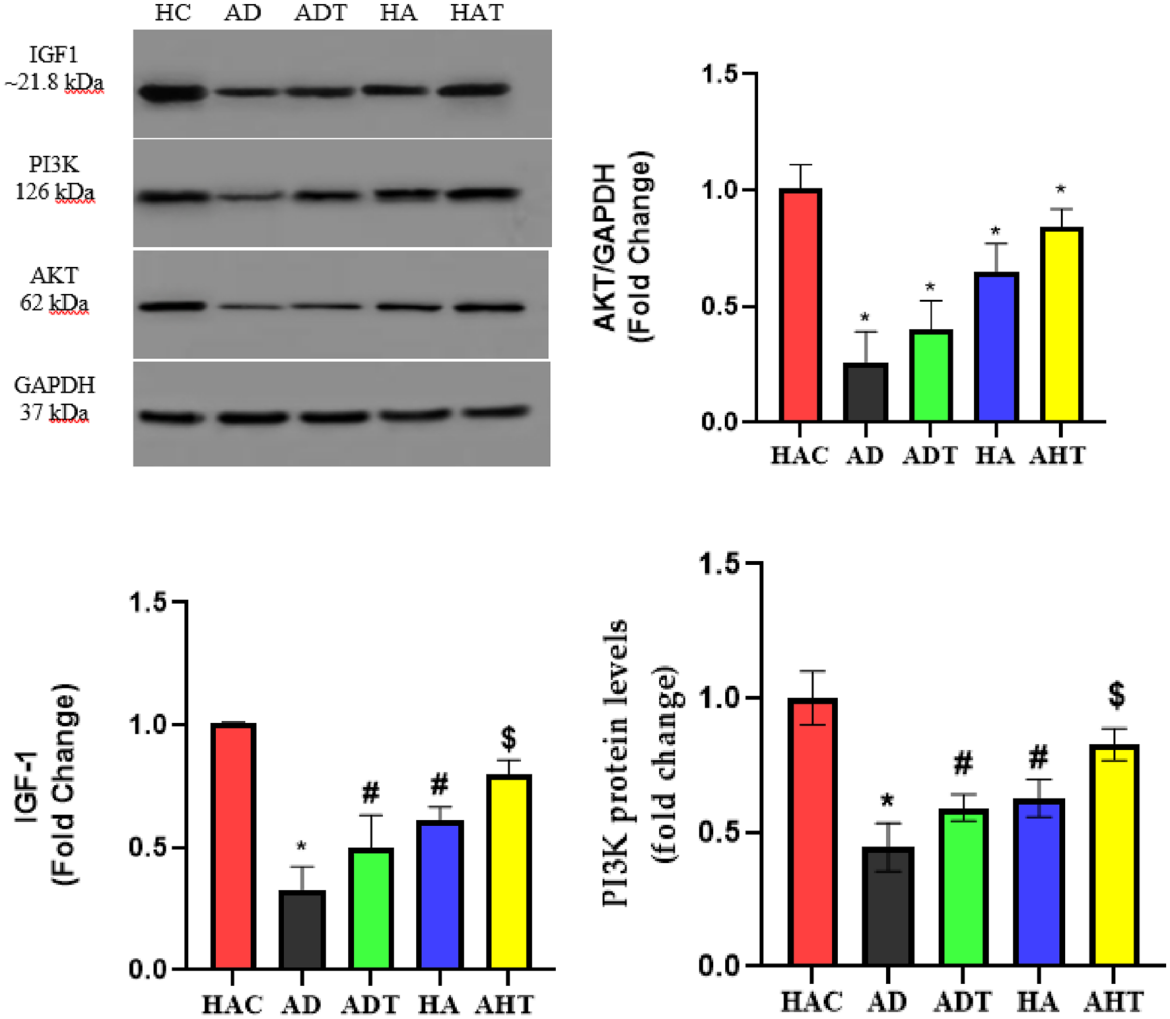
Evaluation of protein content using western blot analysis. (**A**) Expression of IGF-1, PI3K and AKT proteins in heart tissue of rats in different groups. **B**–**D**) The relative amount of protein to GAPDH levels by image J software. Asterisk Significant difference with all groups (P < 0.001). Hash Significant difference between ADT and HA groups with other groups (P < 0.05).

The Fig. 7 illustrates the amounts of IGF-1, PI3K, and AKT proteins in the heart tissue of rats in different groups. The findings indicated that the content of IGF-1, PI3K, and AKT proteins in the AD group significantly decreased compared to the HAC group (P < 0.001). Significant differences were also observed between the AD group and the ADT group (P < 0.05). Furthermore, the content of these proteins in the HA group was significantly lower than the HAC group (P < 0.05). The study also demonstrated that the increase in the content of these proteins in the AHT group was significant compared to the HA group (P < 0.05), while no significant difference was observed in the content of these proteins between the AHT and the HA groups (P > 0.05).

## Discussion

The current study aimed to investigate the effect of HIIT on histological changes and protein content related to hypertrophy and apoptosis of heart tissue in aged rats with T2DM. The findings indicated that ADT group undergoing HIIT had a smaller reduction in body weight and a significant decrease in blood glucose compared to the AD group. These results are consistent with previous studies by Rami et al.^6^, Cassidy et al.^30^, and Adams^31^, which demonstrated that HIIT can lead to a decrease in blood glucose in T2DM groups compared to control groups. Regarding histological changes, the study found that cross-sectional area enlargement of cardiomyocytes occurred in the hearts of old rats, indicating pathological hypertrophy, which HIIT managed to control by reduction of pathological hypertrophy and increase of physiological hypertrophy in the aging diabetes training group. This outcome aligns with Rami et al.’s study^6^. Moreover, the heart of old rats showed a significant increase in interstitial fibrosis, which HIIT successfully lowered by reducing collagen accumulation and interstitial fibrosis in the heart tissue of the ADT group. The study also revealed that elderly diabetic rats had fat accumulation in cardiomyocytes, which HIIT significantly reduced in the cardiomyocytes of the ADT group. The diabetic heart relies heavily on fatty acids as an energy source instead of glucose, leading to increased oxidative stress and mitochondrial dysfunction. Impaired fatty acid oxidation (due to mitochondrial dysfunction) can result in the accumulation of fat droplets and lipotoxicity in the myocardium^32^. PE plays a crucial role in improving insulin sensitivity (by upregulating PKC expression), increasing GLUT-4 expression, enhancing glucose uptake, and reducing oxidative stress^33,34^. These effects enable cardiomyocytes to eliminate accumulated fats more efficiently. Additionally, exercise-induced elevations in PGC-1, along with peroxisome proliferator-activated receptor α (PPARα), regulate fatty acid import, storage, and oxidation in the heart^35^. PGC-1a can also independently stimulate Akt activation, as demonstrated by the promotion of the Sirt1/PGC-1α/Akt signaling pathway in cardiomyocytes of myocardial infarction rats after four weeks of running training^36^. The interconnected relationship between AMPK-Sirt1 and PGC-1α helps regulate cardiomyocyte mitochondrial metabolism. Treadmill running promotes AMPK/PGC-1α signal transduction, which decreases the accumulation of reactive oxygen species in the rat myocardium^37^. Moreover, high-intensity interval training has the potential to promote autophagy^38^. Although further research is needed, a specific form of selective autophagy called lipophagy (which is reduced in T2DM) may be one of the potential mechanisms involved in removing fat droplets in cardiomyocytes^39^. Additionally, the examination of protein changes indicated that the content of IGF-1, PI3K, and AKT proteins in the AD group was significantly lower than the HAC group, but HIIT led to a significant increase of these proteins in the ADT group.

In the first part, the current study examined changes related to weight and blood glucose. The findings revealed that T2DM can lead to weight loss, which is consistent with previous studies conducted by Ruissen et al.^40^, Lewandowska et al.^41^, Rami et al.^6^, and Ferreira et al.^42^. Further, the study established that diabetes can cause a significant increase in blood glucose, which is in line with the studies conducted by Rami et al.^6^, Gabbay et al.^43^, Bergenstal et al.^44^, and Ripsin et al.^45^. In this context, Rami et al.^6^ revealed in their study that HIIT can reduce blood glucose in diabetic groups in comparison to control groups. Therefore, it can be inferred that HIIT can be a promising strategy to mitigate blood glucose levels in elderly individuals with diabetes. The incorporation of HIIT into routine physical activity regimens can be a pragmatic approach to managing diabetes-related weight gain and blood glucose levels in the elderly population.

In the second part of the present study, the histological changes of the heart tissue in AD rats were examined. The results indicated an increase in the cross-sectional area of cardiomyocytes in the heart tissue of AD rats. This finding suggests that diabetes can be a significant contributing factor to pathological hypertrophy of the heart. In agreement with the present study’s findings, Rami et al.^6^, Novoa et al.^46^, and Lu et al.^47^ confirmed that diabetes increases the cross-sectional area and thickness of cardiomyocytes. This increase is one of the primary disorders related to cardiovascular diseases, which can lead to death in old age^48^. Additionally, these changes in the heart can cause high blood pressure diseases. Regarding studies related to HIIT and changes in cardiomyocytes of diabetic rats, we recent findings have demonstrated that HIIT can significantly reduce the cross-sectional area of cardiomyocytes in the heart tissue of rats with type 2 diabetes^6^. In a study conducted by Novoa et al. in^46^, it was noted that diabetes induces cellular hypertrophy of cardiomyocytes as part of the regeneration process in the TDM2 heart. However, intense training was found to reduce this hypertrophy in cardiac cells. The study also revealed that T2DM rats exhibited an increase in cardiomyocytes compared to the control group, but this value decreased in diabetic rats that underwent intense PE. Furthermore, the cross-sectional area of cardiomyocytes in the hearts of T2DM rats increased in comparison to the control group, but decreased in animals that underwent physical training^46^. A study by Cassidy et al.^50^ in, which focused on patients with T2DM using MRI, demonstrated that HIIT could promote positive cardiac remodeling. After 12 weeks of HIIT, patients showed an increase in left ventricular wall mass and end-diastolic blood volume^30^. In 2020, Guqiang et al.^49^ conducted a study investigating HIIT for the treatment of pathological hypertrophy in spontaneously hypertensive rats. The researchers concluded that 8 weeks of HIIT could induce a transition from pathological hypertrophy to physiological hypertrophy. Additionally, it was found to enhance heart function in spontaneously hypertensive rats through the activation of the PI3K/Akt signal transduction pathway^49^. Verboun et al.^50^ demonstrated in their study that HIIT were more effective in inducing physiological hypertrophy and increasing the thickness of heart walls in healthy rats compared to exercises of moderate intensity^50^. Overall, the results of the present study, in conjunction with previous research, indicate that HIIT can effectively control and reduce pathological hypertrophy caused by diabetes. Additionally, in healthy mice, these exercises can promote physiological hypertrophy These results align with the findings of other studies^46^. Therefore, incorporating HIIT into routine PE regimens may be a promising approach to mitigating pathological hypertrophy of the heart tissue in elderly individuals with TDM2. Such an approach may also help to reduce the risk of cardiovascular diseases associated with diabetes-related changes in the heart. Aligned with the investigation of histological alterations, the current study unveiled a noteworthy augmentation in interstitial fibrosis within the heart of elderly rats. It has been suggested that cardiac fibrosis associated with diabetes may be the primary cause of mortality due to its ability to induce heart failure and increase the incidence of arrhythmic events^51^. Additionally, the cellular and molecular alterations associated with cardiac fibrosis suggest that it contributes to diastolic dysfunction and arrhythmogenesis, and that neural and inflammatory pathways may activate diabetic fibroblasts^51^. Regarding the assessment of the impact of HIIT, the results of the present study have demonstrated that HIIT could potentially lead to a reduction in cardiac fibrosis in the aging diabetes training group. These findings are in line with the results of previous research conducted by Rami et al.^6^, Novoa et al.^46^, and Lu et al.^47^. Furthermore, Rami et al.^6^ reported that HIIT significantly reduced cardiac fibrosis in type 2 diabetic rats.

In the histological analysis, the current study has demonstrated an elevation in the accumulation of lipid droplets in the cytoplasm of cardiomyocytes within the elderly diabetic group. Furthermore, it has been revealed that cardiac tissue fat in diabetic rats can be a primary contributing factor to the increase in cardiovascular diseases^52,53^. The accumulation of fat within the cytoplasm of cardiomyocytes can also result in a reduction in immune system function and an increase in the infiltration of inflammatory cells into the heart’s cardiomyocyte^52,53^. The findings of the present study are consistent with the results of Noyes et al.^52^, Colantuoni et al.^53^ and Konwerski et al.^54^. In regards to the exercise intervention, the outcomes of the current research indicate that HIIT may lead to a reduction in lipid droplets within the cytoplasm of cardiomyocytes and decrease the infiltration of inflammatory cells.

In the final section, the present study investigated the levels of IGF-1, PI3K, and AKT proteins in the heart tissue of rats. The results demonstrated a significant reduction in the content of IGF-1, PI3K, and AKT proteins in the elderly diabetic group in comparison to the healthy control group. The IGF-1/PI3K/AKT signaling pathway is present in various organs within the body and plays a crucial role in multiple physiological functions^12^. Additionally, it has been established that IGF-1 plays a crucial role in maintaining the homeostasis of cardiac physiology, and negative alterations in IGF-1 levels can lead to cardiac complications such as atherosclerosis, inflammation, vasodilation, cardiac apoptosis, and autophagy^11,12,14^. Corresponding to the adverse changes in IGF-1 within the heart, negative changes in PI3K and AKT can also lead to atherosclerosis in the heart^11,12,14^. IGF-1 has been previously shown to have an inverse correlation with CRP in studies^55^, and HIIT has been found to be more effective than other exercise modalities in reducing CRP levels^56,57^. Moreover, hyperglycemia directly inhibits the production of IGF-1^58^, and chronic hyperglycemia can suppress the secretion of growth hormone (GH), which in turn reduces IGF-1 production since GH stimulates its synthesis^59^. Additionally, in diabetic rats, high-intensity interval training has been found to enhance diabetic cardiomyopathy through the miR-1-mediated suppression of cardiomyocyte apoptosis. MiR-1 acts as a negative regulator of IGF-1, meaning it suppresses its production. Both conventional and HIIT protocols significantly decreased miR-1 expression in the left ventricular tissue of diabetic rats. Furthermore, the HIIT protocol exhibited a greater reduction in miR-1 compared to the conventional protocol. The authors proposed that this decrease in miR-1 levels contributed to increased IGF-1 levels and improved cardiac function^60^. In addition to the IGF-1 pathway, exercise can also activate intracellular PI3K through mechanotransduction, leading to the aggregation of Neuregulin-1 and the activation of ErbB2/ErbB4 tyrosine kinase receptors. These receptor activations are crucial for promoting cardiomyocyte proliferation, differentiation, and regeneration^35^. The results of the HIIT intervention in the present study revealed that PE could lead to positive alterations in the content of IGF-1, PI3K, and AKT proteins.

One of the strengths of the present study lies in its examination of both histological and molecular changes, providing detailed insights into the alterations following the implementation of HIIT. Additionally, the investigation of signaling pathways and the use of various color markers were other strengths of this study that substantiated the efficacy of HIIT. On the other hand, it is important to acknowledge the limitations of our study. One such limitation is the non-utilization of other laboratory methods like immunohistochemistry and TUNEL assay, as well as cardiac function tests like ECG to investigate the process of apoptosis and other tissue changes and cardiac dysfunction. These tests were not conducted due to the unavailability of laboratory facilities. In future research, it is recommended that researchers carefully examine the changes in the heart tissue of diabetic samples using these methods, in order to gain a more comprehensive understanding of the effects of HIIT on cardiac function and tissue changes in elderly individuals with T2DM.

## Conclusion

Ultimately, the findings of the present study suggest that HIIT can be a viable approach for enhancing both histological and physiological changes in elderly individuals with T2DM. Although HIIT has been employed in medical rehabilitation, the suitable PE protocol for the prevention of cardiac events remains under debate. The varied strategies employed in prior studies also make cross-comparative analysis challenging. Moreover, the outcomes of our investigation represent only a fraction of the cellular, molecular, and histological interactions present within the heart tissue of T2DM rats. This study does not provide conclusive, robust, and comprehensive findings on PE-induced events and their effects on diabetic samples. Nevertheless, it is clear that further research is required to investigate the impact of intense PE on both healthy and sick samples. By identifying other factors involved in cardiomyocyte homeostasis, researchers can develop a sustainable, non-pharmacological approach to aid patients with diabetes and other diseases. In general, the current study demonstrated that HIIT can be appropriate for elderly individuals with T2DM. To validate this finding, a systematic study and meta-analysis were conducted, revealing that HIIT is a safe protocol for elderly individuals with T2DM^61^. As a result, HIIT can be considered suitable due to their inherent nature of producing positive physiological effects within a short period of time. However, it is recommended that further research be conducted in future studies to corroborate these findings.

### Supplementary Information

Supplementary Information 1.

Supplementary Information 2.

Supplementary Information 3.

## Data Availability

The datasets used and/or analyzed during the current study are available from the corresponding author upon reasonable request.

## Abbreviations

T2DM: Type 2 diabetes mellitus
CVD: Cardiovascular disease
HAC: Healthy adult control
HA: Healthy aging
AD: Aging diabetes
AHT: Aging health training
ADT: Aging diabetes training
HIIT: High-intensity interval training
IGF-1: Insulin-like growth factor 1
PI3K: Phosphoinositide 3-kinases
AKT: Protein kinase B
PE: Physical exercise
MF: Myocardial fibrosis
HF: Heart failure
CAD: Coronary artery disease
AF: Atrial fibrillation
PAD: Peripheral artery disease
IRS-1: Insulin receptor substrate
MDT: Moderate-intensity training
