# Supplementary material for: Highlighting the effects of high-intensity interval training on the changes associated with hypertrophy, apoptosis, and histological proteins of the heart of old rats with type 2 diabetes
Source: Sci Rep. 2024 Mar 26;14:7133. doi: 10.1038/s41598-024-57119-6 (PMC10966008; doi:10.1038/s41598-024-57119-6)
Supplement: Supplementary file 1 — Supplementary Information 1. [file 41598_2024_57119_MOESM1_ESM.docx]

Supplementary Material

# Solutions needed to prepare STZ

## Preparation method of 0.1 M citrate buffer

Dissolve 50 ml of distilled water in 1.47 grams of sodium citrate and adjust its pH to 4.5 with acetic acid.

## How to prepare citric acid

0.51 g of citric acid monohydrate (Sigma, USA) was dissolved in 50 ml of distilled water and kept at room temperature.
